# Supplementary material for: Serum Leptin Levels, Nutritional Status, and the Risk of Healthcare-Associated Infections in Hospitalized Older Adults
Source: Nutrients. 2022 Jan 5;14(1):226. doi: 10.3390/nu14010226 (PMC8747117; doi:10.3390/nu14010226)
Supplement: Supplementary file 1 [file nutrients-14-00226-s001.zip › nutrients-1507179-supplementary.pdf]

Table S1. Sensitivity analysis. Multivariate analyses of women with at least one healthcare-associated infection vs. women without infections (n = 168).

	OR [95%CI]	*p* Value
CIRS-G score [a]	1.54 [1.02–2.32]	0.039
Invasive procedure	6.38 [2.70–15.09]	<0.001
Naïve CD8+ T-cells (CD45RA+CD62L+)% [b]	0.68 [0.47–0.99]	0.048
Serum leptin level 4.2 μg/L [c]	2.25 [1.02–5.11]	0.045

OR—odds ratio; CI—confidence interval; CIRS-G—Cumulative Illness Rating Scale, Geriatric. The multivariate analysis was adjusted for all variables of the model. The odds ratios [95%CI] estimated using logistic regression models are quoted for [a] a 1-SD increment in the log transformed values or [b] a 1-SD decrease in the log transformed values. [c] The leptin level was dichotomized according to the lowest quartile. 4.2 μg/L is the lowest quartile of serum leptin among women.
